# Supplementary material for: Electrically Conductive Nanocomposites Composed of Styrene–Acrylonitrile Copolymer and rGO via Free-Radical Polymerization
Source: Polymers (Basel). 2020 May 27;12(6):1221. doi: 10.3390/polym12061221 (PMC7362241; doi:10.3390/polym12061221)
Supplement: Supplementary file 1 [file polymers-12-01221-s001.pdf]

Electrically Conductive Nanocomposites Composed of Styrene-Acrylonitrile Copolymer and rGO via Free-Radical Polymerization

Eun Bin Ko[1], Dong-Eun Lee[2,*] and Keun-Byoung Yoon[1,*]

[1]School of Chemistry & Chemical Engineering, Anhui University of Technology, China

[2]Department of Polymer Science and Engineering, Kyungpook National University, Korea

[3]Display Nanomaterials Institute, Kyungpook National University, Korea

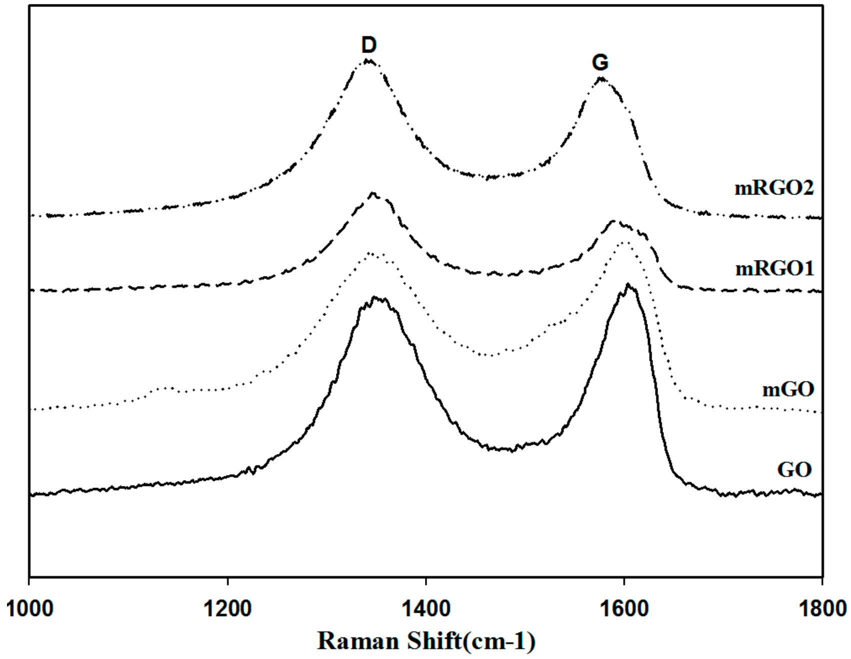

Figure S1. Raman spectra of GO, mGO, mRGO1 and mRGO2.

Raman spectroscopy was performed to determine the ordered and disordered carbonaceous materials, and the results are shown in Figure S1.

It is well known that graphene sheets exhibit three prominent peaks; D-band at 1313 cm^{-1}, G-band at 1577 cm^{-1}, and 2D-band at 2641 cm^{-1}. The D-band at 1313 cm^{-1} is in the out of plane breathing mode, which originates from defects in graphene. The G-band at 1577 cm^{-1}, corresponding to the in-plane bond stretching motion of the sp^2 carbon structure, is attributed to the aromatic domains [1]. The presence of defects shifts the G-band to higher frequencies for GO, mGO and mRGOs. The G-band of GO appeared at 1603 cm^{-1}, which is higher than that for pristine graphene, because the oxygen-containing groups act as defects on the surface of graphene. After modification and reduction of GO, the red-shift of the G-band occurred

close to that of the G-band of graphene sheets, indicating that the conjugated network of graphene recovered to some extent [2]. The D/G band intensity ratio (I_D/I_G) is an indication of the defect-like amorphous domains. The I_D/I_G values, which were 1.37 and 1.13 for mRGO1 and mRGO2, respectively, were higher than that of GO (0.98). This is explained by the fact that the number of aromatic domains increased and the size of the sp^2 carbon domain decreased after the reduction step [3].

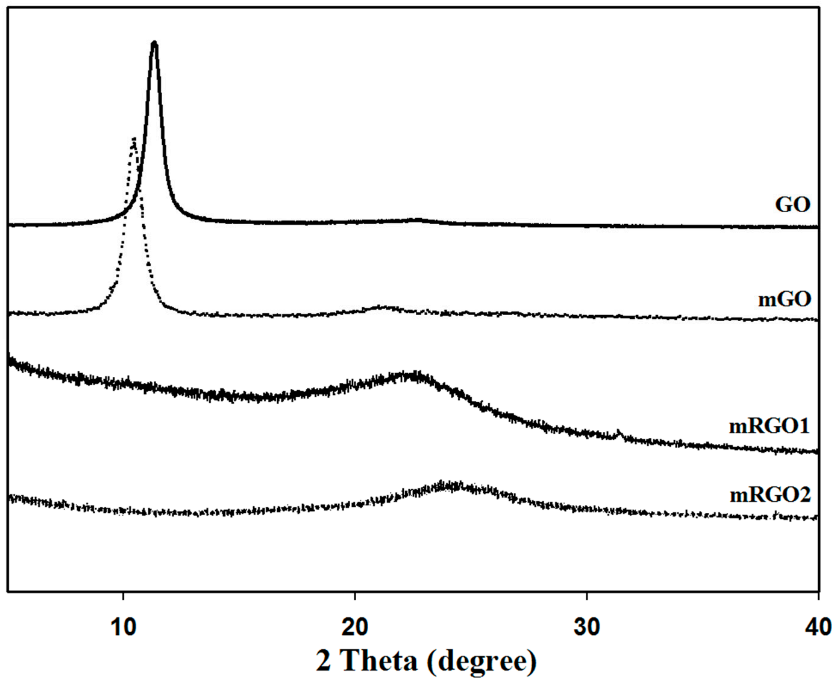

Figure S2. XRD patterns of GO, mGO, mRGO1 and mRGO2.

XRD measurements were obtained to determine the changes in the interlayer distance. Figure 3 shows the XRD patterns of GO, mGO and mRGOs. The diffraction angle of GO was approximately 11.3°, which corresponds to an interlayer distance of approximately 0.78 nm. After the modification of GO with MPS, the diffraction angle decreased from 11.3° to 10.4°,

corresponding to an increase in the interlayer spacing from 0.78 to 0.85 nm. However, these peaks disappeared after the reduction of mGO. These phenomena indicate that MPS is bonded on the surface of GO successfully and is capable of enlarging the interlayer distance between GO sheets. For mRGO1 and mRGO2, a new broad peak can be observed at 22~24°, which are close to that of pristine graphene nanosheet (25°). This indicates that the mRGOs sheets were exfoliated into a few layers, which resulted in a new sp^2 carbon structure different from that of graphene nanosheet [4].

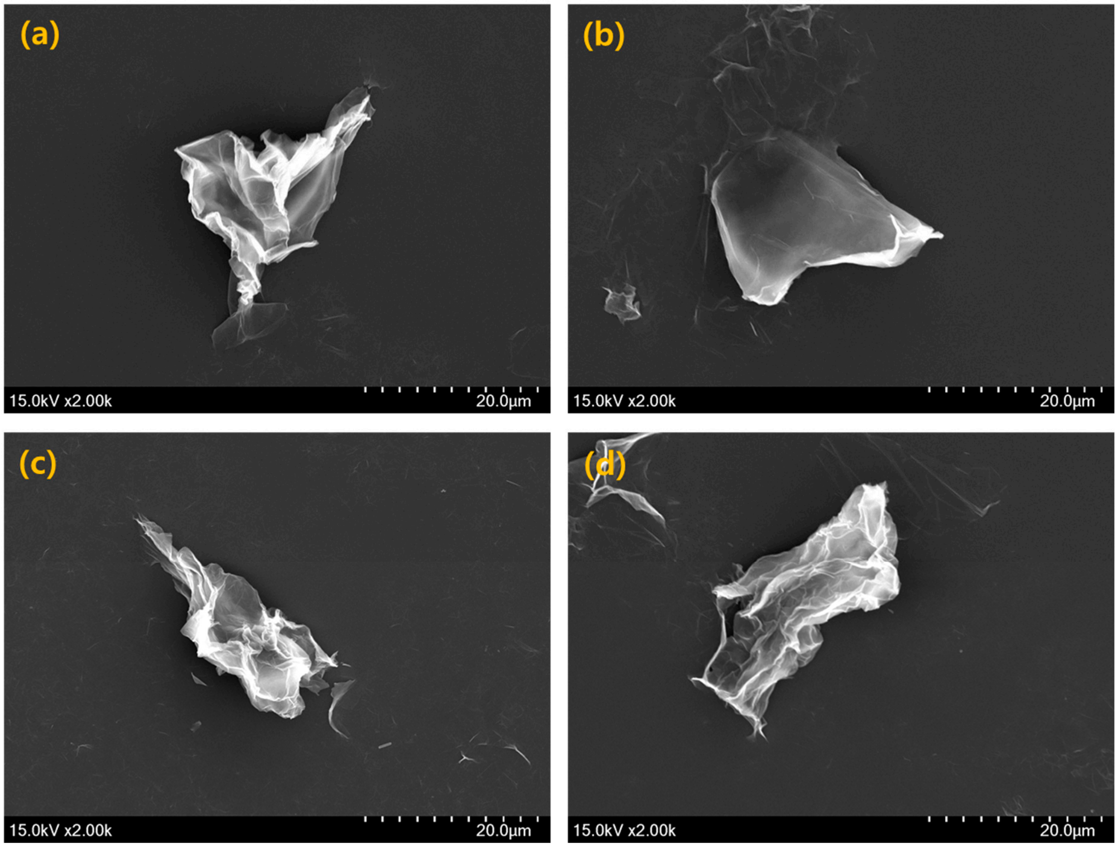

Figure S3. SEM images of (a) GO, (b) rGO, (c) mGO, and (d) mRGO2.

SEM samples of GO, rGO, mGO and mRGO2 were prepared by freezed dried. Figure S3 shows SEM images of exfoliated GO, mGO and mRGO2, which exhibits the typical wrinkle morphology. Meanwhile, the surfaces of mGO and mRGO2 were observed with more wrinkles than that of GO. The rGO was free of wrinkles on the surface and had multiple layers. This is because the layer was restacked during the solvothermal reduction process.

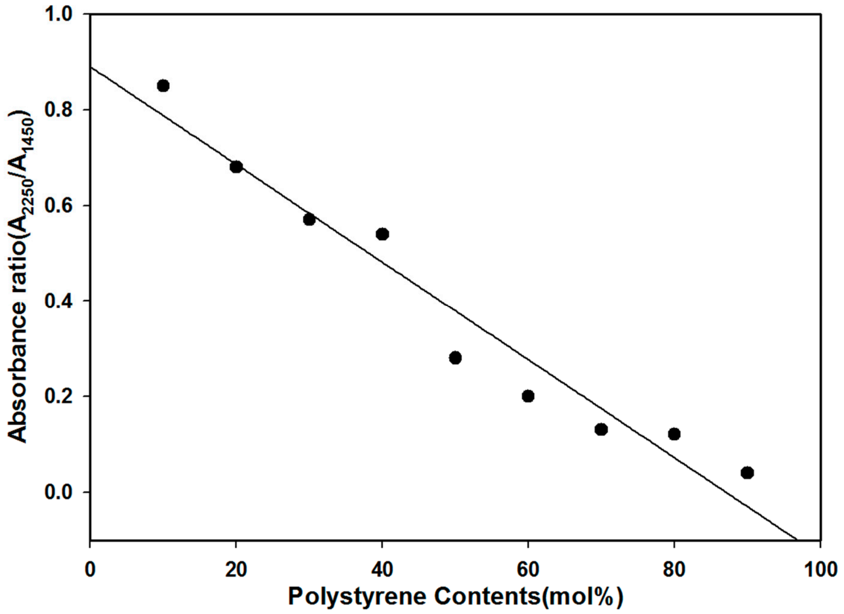

Figure S4. Relationship between absorbance ratio and styrene contents of styrene-acrylonitrile copolymers.

1. Ferrari, A.C.; Meyer, J.C.; Scardaci, V.; Casiraghi, C.; Lazzeri, M.; Mauri, F.; Piscanec, S.; Jiang, D.; Novoselov, K.S.; Roth, S.; Geim, A.K. Raman spectrum of graphene and graphene layers. *Phys. Rev. Lett.*, **2006**, 97, 187401.
2. Fang, M.; Wang, K.; Lu, H.; Yang, Y.; Nutt, S. Covalent polymer functionalization of graphene nanosheets and mechanical properties of composites. *J. Mater. Chem.* **2009**, 19, 7098-7105.

3. Paredes, J.I.; Villar, R.S.; Solis, F.P.; Martinez, A.A.; Tascon, J. atomic force and scanning tunneling micsroscopy imaging of graphene nanosheets derived from graphite oxide. *Langmuir*, **2009**, 25, 5957-5968.

4. Lerf, A.; Buchsteiner, A.; Pieper, J.; Schöttl, S.; Dekany, I.; Szabo, T.; Boehm, H.P. Hydration behavior and dynamics of water molecules in graphite oxide. *J. Phys. Chem. Solids* **2006**, 67, 1106-1110.
